# Increase in abundance and decrease in richness of soil microbes following Hurricane Otto in three primary forest types in the Northern Zone of Costa Rica

**DOI:** 10.1371/journal.pone.0231187

**Published:** 2020-07-30

**Authors:** William D. Eaton, Katie M. McGee, Kiley Alderfer, Angie Ramirez Jimenez, Mehrdad Hajibabaei

**Affiliations:** 1 Biology Department, Pace University, New York, NY, United States of America; 2 Department of Integrative Biology, Biodiversity Institute of Ontario, University of Guelph, Guelph, ON, Canada; UNAM, MEXICO

## Abstract

Little is known of how hurricane-induced deposition of canopy material onto tropical forest floors influences the soil microbial communities involved in decomposition of these materials. In this study, to identify how soil bacterial and fungal communities might change after a hurricane, and their possible roles in the C and N cycles, soils were collected from five 2000 m^2^ permanent plots in Lowland, Upland and Riparian primary forests in Costa Rica 3 months before and 7 months after Hurricane Otto damaged the forests. The soil Water, inorganic N and Biomass C increased and total organic C decreased Post-Hurricane, all of which best predicted the changes in the Post-Hurricane soil microbial communities. Post-Hurricane soils from all forest types showed significant changes in community composition of total bacteria, total fungi, and five functional groups of microbes (i.e., degrading/lignin degrading, NH_4_^+^-producing, and ammonium oxidizing bacteria, and the complex C degrading/wood rot/lignin degrading and ectomycorrhizal fungi), along with a decrease in richness in genera of all groups. As well, the mean proportion of DNA sequences (MPS) of all five functional groups increased. There were also significant changes in the MPS values of 7 different fungal and 7 different bacterial genera that were part of these functional groups. This is the first evidence that hurricane-induced deposition of canopy material is stimulating changes in the soil microbial communities after the hurricane, involving changes in specific taxonomic and functional group genera, and reduction in the community richness while selecting for dominant genera possibly better suited to process the canopy material. These changes may represent examples of taxonomic switching of functionally redundant microbial genera in response to dramatic changes in resource input. It is possible that differences in these microbial communities and genera may serve as indicators of disturbed and recovering regional soil ecosystems, and should be evaluated in the future.

## Introduction

The occurrence of tropical hurricanes are thought to likely be increasing in frequency in the future [1, 2]. These disturbances alter the forest vegetation composition and productivity through tree damage [3]. Hurricanes also cause rapid deposition of significantly large amounts of leaf litter and woody debris from the canopy to the forest floor [4–7], which then results in short to long-term changes to the forest structure and forest ecological processes [4]. These processes are critical to forest recovery, and are associated with both the rapid increase in the carbon (C) components and nitrogen (N) nutrients [8–11] and the subsequent influences to the soil microbiota involved in the dynamic processes of the C and N cycles [12, 13]. However, despite the known critical ecological roles of soil microbes, there is a knowledge gap in understanding the composition of these biota in tropical soils, and the specific roles they play in the biogeochemical cycling, especially in recovery after forest disturbance. Increasing our understanding of these biota would be beneficial in assessing the impacts that land disturbances, including hurricanes, have had on tropical soils, and the efficacy of different restoration practices used in ecosystem recovery.

Soil bacteria are involved in C and N cycle processes associated with early stages of soil succession or recovery from disturbance, such as decomposition of less complex organic C, N-fixation, and ammonium oxidation [14, 15]. Major forest disturbances, such as hurricanes, that alter the quantity and quality of plant resource inputs into the forest soil, change the concentrations of critical soil substrates, or modify the soil water, pH or temperature levels in the soil will influence the composition of the soil bacterial communities and the types and rates of their metabolic activities that involve utilization and processing of various C and N components [16–18]. However, due to their critical role in the C and N cycle processes, these same bacterial groups are also expected to be the first biota to re-establish in the soils during the early recovery stages following disturbances. The soil fungal community, also altered by forest disturbances, are thought to be more important in later soil successional or recovery than bacteria. This is due to their abilities to decompose the complex organic substrates that develop during earlier stages of soil succession or recovery more efficiently than bacteria, leaving behind more recalcitrant residues, and enhancing the soil organic C matter [19–21]. These fungi require the N and C inputs from the earlier bacterial activities in order to produce the enzymes necessary for activities such as wood rot, degradation of lignin, and decomposition of other complex forms of organic C [19–22].

Studies on the effects of hurricane-induced litter fall, changes in soil moisture, and enhanced soil nutrients on soil processes (especially decomposition) have shown the patterns observed are a function of the characteristics of the specific hurricane, and those of the forest before the hurricane hit, making it difficult to predict specific effects from the hurricane-induced dropping of canopy material [8]. Several studies of the influences of actual hurricanes, rather than simulations (i.e., Hurricane Jova and Patricia that hit Mexico) found that the deposition of canopy material stimulated pulses of N in the soils, in particular ammonium and nitrate, and, at least in one case, temporarily also increased the decomposition rates [23, 24]. However, not surprisingly, much of the leading work on the impacts of hurricanes on soils before and after the event have relied on results from hurricane simulation experiments in which canopy components are cut and dropped to the forest floor [6, 9, 16, 25, 26], which leads to the important question of “what happens under real conditions and are they different?”. Clearly, there is much work to be conducted, but doing work in the same forest location before and after a hurricane is truly a serendipitous opportunity. This paper describes just such a serendipitous opportunity to study the effects of a hurricane on the same tropical forest soils before and after a hurricane in three different habitats within a Costa Rican Tropical forest.

Lugo [8] suggests that there are shorter-term Immediate Effects that a hurricane has on a tropical forest of 0 to 3 years, and possible longer-term Intermediate Responses of 0 to 20 years. In hurricane simulation field experiments, the canopy material dropped to the forest floor stimulated nutrient pulses of more labile forms of C, N and phosphorus derived from the younger leaf litter from the canopy, which were generally were processed in the first year post-deposition of material [4, 6, 9, 10, 27, 28]. In addition, one of the more immediate effects due to the loss of canopy cover was an increase in soil moisture, which is believed to be associated with altered nutrient cycle dynamics and, in, particular, decomposition, presumably as it both extracts organic C from woody debris and also alters the soil microbial communities associated with decay and decomposition processes [9, 25, 29]. Liu et al ([10]) also showed the initial pulse of labile organic C and increased soil nutrient turnover rates in the forest soils after hurricane simulation lasted from about the first week to about 2 years post-canopy material deposition, which also corresponded to an increase in the soil microbial biomass that lasted from week 1 until at least 120 weeks after deposition of the canopy material. After this time period, the hurricane-induced soil moisture changes and deposition of course woody debris from the canopy was associated with increased immobilization of the organic matter, and C and N components, resulting in a decreased availability of these for the food web [11, 19, 28–31], and slowing down decomposition activities in the forest soils [9].

Some of these hurricane simulation experiments correlated the changes in the forest floor canopy leaf litter and woody debris, and the pulses of C and N with potential differences in overall soil microbial community structure between the forest floor soils with the canopy material deposited on them as compared to control sites [14, 26], however, no information was presented on any specific microbial taxa or functional groups possibly involved. That said, these are extremely important studies that have helped to develop some basic information on the potential activity of different soil processes that might occur post-hurricane, and provide the opportunity for suggestions as to which soil microbial taxa or functional groups might be affected by the additional canopy material. Cantrell et al. [26] found differences in the overall bacterial and fungal community structure, bacterial richness, and bacterial and fungal diversity between the soils with leaf litter and woody debris deposited on them, compared to untreated forest floor soils. However, as this involved using ester link fatty acids methyl ester (EL-FAME) and terminal restriction fragment length polymorphism (TRFLP) analyses, no taxa or specific functional groups were identified. Lodge et al [25] found differences in the amount of fungal hyphal connections present in leaf litter between these treated and untreated soils using visual observations. Others have shown an increases in soil C, microbial biomass C and N between sites with experimentally deposited canopy material, and also between sites by season, and beneath and in close proximity to the larger woody debris from the experimental vegetation deposition as compared to the soil more distant to the debris [9, 26]. Another study showed there was an initial (1 week post canopy experimental deposition) to longer-term (120 weeks post-deposition) increase in microbial biomass C occurring in the leaf litter on the forest floor during the experiment [10]. In a review by Lugo [8], the idea was presented that the extensive defoliation of young leaf material and its deposition on the forest floor would enhance early N cycle activity, and perhaps result in changes in the associated soil microbial communities McDowell et al [32] found that riparian soils had increased levels of nitrate and ammonium following hurricane-induced deposition of leaf litter, which was absorbed from the soils by the vegetation within the first few years. However, no information was provided on changes in the microbial communities associated with these changes for any of these studies.

What appears to be missing from these works is the classic “before and after” study design in which forest soils are analyzed for differences in C and N nutrients, microbial biomass C, fungal and bacterial community composition of the overall genera, and in the composition of different important microbial functional group taxa within the exact same forest plots before and after a naturally occurring hurricane, instead of a simulation. This is not surprising, as, in general, this would require a serendipitous event for it to happen On November 24, 2016, Hurricane Otto hit the forests in the Northern Zone of Costa Rica providing just such a serendipitous opportunity to conduct a before and after study in the exact same long-term forest plots. Permanent plots established previously in three primary forest types within the Maquenque National Wildlife Refuge in the Northern zone of Costa Rica, an Upland Forest Type, Lowland Forest Type, and Riparian Forest Type, and used for various studies [33–38], with soil samples collected for a project in the summer of 2016. Upon arrival at the sites in summer 2017, we found that all plots in all three forest types had been hit hard by Otto, significantly decreasing the percent canopy cover by about 40 to 70%, and littering the forest floor with young leaf litter and large amounts of woody debris. This provided us with the opportunity for the classic before and after study we report on in this article, in which the soil microbial communities and C and N cycle, and Biomass C metrics were compared within 3 primary forest habitats before and only y months after Hurricane Otto, within the exact same long-term plots. This is still within the timeframe as described for analysis of evidence of early effects of a hurricane [8, 10].

The goal of this study was to determine how Hurricane Otto influenced the soil ecosystem conditions within the first 7 months after causing damage to the tropical forest. Our aim was to determine how soil C and N factors, Biomass C, and associated bacterial and fungal community compositions differed before and after the hurricane caused serious damage to three different types of primary tropical forests (Upland, Lowland, and Riparian) within the Northern Zone of Costa Rica. For this work, three broad questions were asked: (1) Are there differences in the total bacterial and fungal community compositions, and the compositions of the communities of several critical microbial functional groups or genera important to the N and C-cycle, and organic C-use efficiency [39, 40] in the different forest habitat soils before and after the hurricane? (2) Are there differences in the soil temperature, % water, C and N-cycle metrics, C biomass development, organic C-use efficiency between the three types of primary forest habitat soils before and after the hurricane? (3) Are there specific soil environmental variables that appear to shape, or drive the soil microbial community structures? Based on the previous literature [4–11, 22–28], we predicted that the hurricane would open the canopy of all three forest types, depositing fresh leaf litter and excessive woody debris on the forest floor, and exposing the forest floor more directly to precipitation and increasing the % soil water. Collectively, these would enhance soil microbial activity during this period of time 7 months Post-Hurricane in the soil ecosystems of all three forest habitat types resulting in: a) increased levels of NO_3_^-^, NH_4_^+^, along with an increase in abundance and decrease in richness of N-cycle-related bacterial functional groups and genera; b) increased levels of soil Biomass C, Microbial Quotients (Biomass C/TOC), and an increase in the abundance and decrease in richness of bacterial and fungal functional groups associated with the decomposition of more complex forms of organic C, such as polyaromatics, hemicelluloses, lignin, etc.; c) a decrease in the richness of total bacterial and total fungal genera, as these communities would become more specialized to process the canopy-deposited material on the forest floor; and d) these differences in microbial community composition would be best explained by differences in the levels of soil inorganic N and Biomass C levels before and after the hurricane.

## Methods

### Study site

In 2001, the Costa Rican Ministry of the Environment and Energy created the San Juan-La Selva Biological Corridor (SJLBC) to connect six protected areas into a single integrated biological unit of 1,204,812 ha. for habitat connectivity and protection of biodiversity in the Northern Zone ecosystems [41]. The Maquenque National Wildlife Refuge (MNWR; 10^o^27’05.7”N, 84^o^16’24.32”W) was established in 2005 by the Costa Rican government to protect over 50,000 ha. of humid Atlantic lowland primary and secondary forests and other diverse ecosystems within the SJLBC. The MNWR is the core nucleus of the SJLBC as it conserves the highest percentage of forest cover and contains the most valuable habitats for biodiversity [40]. The mean annual temperature of the MNWR is 27°C, mean annual rainfall is 4300 mm, and the dominant soil type is oxisols [42]. The habitats within the MNWR used for this project were: (a) an Uplands Primary Forest, b) a Lowlands Primary Forest, and c) a Riparian Primary Forest. All 3 forests are connected as part of a humid tropical primary rainforest within the MNWR, and all are considered to have oxisol soils. The names of the three habitat types are used by local guides to differentiate the sub-types of forests. The Uplands Forests are on hills, with slightly more sand in the soils resulting a little better drainage than the other two habitats. The Lowland and Riparian Forests are downhill from the Uplands Forests, with greater % clay, and hold more soil water than the Uplands Forest soils. The Riparian Forests are part of the Lowland Forests, but are closer to local unnamed creeks in the region. Unfortunately, no work has been conducted specifically in these sites to compare vegetation species. All sites of the three habitat types were considered by local guides as typically representative of the specific habitat type, and were chosen with their advice based on habitat locations, as either up on hilltops, down in the lower regions > 30 m from a creek, or down in the lower regions < 20 m from the creeks.

### Sample design and soil collection

A bulk soil approach was used for this study to compare soil characteristics at the forest habitat level, eventually using a “before the hurricane” and “after the hurricane” design. In 2010, 5 individual replicate large 50 m x 40 m plots were permanently established in these forest types for future use. The replicate plots were between 100 m and 2 km apart, and established using the standard stratified, block, systematic plot study design used in forestry studies, studies of damaged lands, and restoration ecology studies, as recommended by the American Society of Foresters (ASF; see www.forestandrange.org) and the US Environmental Protection Agency (see EPA 2002 document Guidance on choosing a sampling design for environmental data collection—2002, EPA/240/R-02/005). Soil samples were collected between August 6–10 of 2016, before Hurricane Otto hit the forests of Costa Rica, and again between June 25–30 of 2017, about 7 months (30 weeks) after the hurricane hit the area. The post-hurricane sampling occurred well within the time frame considered by Lugo [8] as the period of “Immediate Effects” on an ecosystem after a hurricane, and also well within the post-hurricane simulation period of time in which Liu et al [10] observed the greatest effects on the soil C metrics.

It is critical to understand the differences in spatial scale when working with soil microbes as compared to “above-ground” organisms in ecology. A distance of several meters to several kilometers is considered a landscape-level separation [43, 44]. Given this and the separation of our soil sampling sites by 100 m to 2 km, the soil microbial communities within the samples collected in this study were separated by landscape-level scales, providing 5 different representative sample plots for each forest type that were true replicates and not pseudo-replicates. To ensure no cross-contamination occurred between plots, thus maintaining the integrity of true replicates, a 7.5 cm x 15 cm x 1.25 cm soil profiler was used to aseptically collect nine soil profile cores of 0–15 cm depth in each plot, using a pre-determined sampling strategy in each plot, with 70% ethanol decontamination of all gloves and tools occurring between plots. Prior to soil collection, the litter layer was carefully removed from the forest floor, exposing the upper organic layer. Soil profiles were collected from the upper humus material, down 15 cm through the organic layer, and into the upper mineral clay region, which typically made up no more than 10–20% of the soil profile core. The nine cores were collected from each plot using a pre-determined sampling plan designed to avoid the plot edges that had been established during cutting of the plot edges (i.e., 10 m, 25 m, and 40 m along a line, and 10 m, 20 m, and 30 m into the plot). All 9 cores for a single plot were combined into a single sterile bag, thus providing six replicate plot samples per habitat type that were used for this study. After collection, using sterile technique, all soil samples were passed through a sterilized 4-mm (standard mesh No. 5) sieve at field moist conditions prior to all downstream analyses. This standard size mesh is commonly used as it retains small gravel pieces, helps to homogenize the soil sample, and allows soil and both microbial cells and fungal hyphae and cells to pass through.

### Possible annual variation in precipitation between sample collection dates

As soil samples were collected in August, 2016 and June, 2017, we needed to determine if any belowground differences observed could be associated with simple seasonal variations in precipitation between these months. The Costa Rican weather service-related sites (such as included in weatherspark.com) identify the rainy season in this region of the Northern Zone as May through November. However, Costa Rica has many microclimates, and with local anecdotal information suggesting rain patterns have changed in the last decade, we analyzed the monthly rain data collected at an unofficial rain gauge station adjacent to the research site for the months of June and August from the 3 years prior to each soil sampling (data not shown). There were no significant differences in the mean levels of precipitation for the months of June and August during this time period (396.17 mm ± 128.6 mm; 333.7 mm ± 137.0, respectively; ANOVA *p* value = 0.596). In addition, although only 1 monthly reading is taken at this site/month, thus, no statistics can be performed, the monthly precipitation reading for August, 2016 at the site was found to be 308 mm, and for June, 2017, it was 336 mm. Given these data, and the national recognition that June and August are about in the middle of the rainy season in the Northern Zone of Costa Rica, it is unlikely that differences in monthly precipitation levels between August 2016 and June 2017, on their own, had a significant influence on any below ground observations made in this study. Thus, any increase in soil water observed at the plots in the Post-compared to Pre-Hurricane soil samples would be due to the hurricane-induced exposed canopy, allowing more rain to reach the soil, and not due to any seasonal variations in precipitations.

### Soil abiotic properties

At the time of sampling, soil temperature was determined. Subsamples (200g) of field moist soil from each sieved soil sample were delivered to the Center for Tropical Agriculture Research and Education (CATIÉ) Laboratories in Turrialba, Costa Rica for determination of soil % water, and C, biomass C and N values. The total organic C (TOC) levels were determined by the dry combustion methods of Anderson & Ingram [45], and the Biomass C determined by standard chloroform fumigation methods, and used to calculate the ratios of Biomass C/TOC to indicate the efficiency of the use of organic C in biomass development [39, 40, 46]. The total N mass (TN) levels were determined by the Kjeldahl method, and NH_4_^+^ and NO_3—_ levels were measured from 2M KCl extracts using the spectrophotometric methods of Alef and Nannapieri [47].

### DNA extraction, sequencing, and bioinformatics

Environmental microbial DNA (eDNA) was extracted from three 0.33g replicate sub-samples for a total of 1g for each soil sample using the MoBio PowerSoil DNA Isolation Kit (MO BIO Laboratories Inc., Carlsbad, CA, USA). The concentration and purity (A_260_/A_280_ ratio) of extracted soil eDNA were determined prior to downstream analyses using a NanoDrop 1000 spectrophotometer (ThermoFisher Scientific, Waltham, MA). All of the following methods are described in detail by McGee et al. [36, 48], with a detailed description provided in S1 Appendix. Briefly, the different eDNA extracts from the soil samples were used for a 2-step PCR amplification of eDNA targeting the nuclear internal transcribed spacer (ITS) ribosomal RNA gene region for fungi [49], and also targeting the v3 and v4 of 16S ribosomal RNA gene region for bacteria and archaea [50]. All generated soil amplicons were sequenced in several Illumina MiSeq runs using a V3 MiSeq sequencing kit (FC-131-1002 and MS-102-3003). The 16S and ITS Illumina-generated sequences were processed using semi-automated pipelines, producing operational taxonomic units (OTUs), which were processed and taxonomically assigned from Phylum to Genus using the Ribosomal Database Project (RDP) classifier v2.12 [51] for bacteria and using the RDP classifier with the UNITE fungal ITS training set for fungi [52]. All DNA sequences were submitted to NCBI SRA (Accession numbers SAMN15405774-79). The number of times a specific DNA sequence (i.e., OTU or genus) appeared in a sample was converted to mean proportion of the sequences per sample [53], hereafter called the MPS. The MPS was determined for each genus within each soil subsample from the different forest types as the number of sequences of an individual genus within a soil subsample, divided by the total number of sequences within that subsample. Subsets of sequences identified to the genus level were categorized into the different functional groups of complex C degrading and lignin degrading bacteria (CCD/Lignin), bacteria producing N ammonium (NH_4_^+^) either from N-fixation or nitrite/nitrate reduction (NH_4_^+^ Producers), complex C degrading/wood rot/lignin degrading fungi (CCD/WRT/Lignin), and ectomycorrhyizal fungi (ECM). Although the ECM are thought to decay complex organic C and recalcitrant soil compounds (see Zak et al [54] for a review), the ECM fungi were kept as a separate category from the CCD/WRT/Lignin fungi. The MPS values of these groups were also determined.

### Data analysis

To address question 1, all MPS values were transformed using a 4th root transformation to account for dominant and rare taxa, as recommended by Anderson et al. [55]. The 4th root transformed data were then converted into Bray-Curtis dissimilarity matrices in PRIMER-E v6 for multivariate analyses. The magnitude of the differences in MPS values between soil samples collected within each type of habitat Pre- and Post-Hurricane were assessed by PERMANOVA, using PRIMER-E v6 and its PERMANOVA+ add-on [55]. Additionally, a Canonical Analysis of the Principal Coordinates (CAP) was performed, using the PERMANOVA+ guidelines [55, 56], to provide a rigorous assessment of the strength of the distinctiveness of the microbial community compositions in the soils before and after the hurricane. Strong differences in the microbiome compositions between the different soil comparisons are indicated by CAP axis squared canonical correlations > 0.7, and moderate differences are indicated by squared canonical correlations > 0.5 to 0.69, less than this are considered weak differences [56]. The same Bray-Curtis matrices were used to calculate the Margalef's richness (d = (S-1)/Log(N)) using PRIMER-E v6 [55] for the total bacterial and fungal genera and for the genera associated with the three different functional groups of interest. The magnitude of the differences in the richness levels before and after the hurricane was assessed by PRIMER-E v6 and its PERMANOVA+ add-on [55]. The magnitude of the differences in the richness levels were also assessed by calculating the Hedge’s *g* Effect Size. Hedge’s *g* for Effect Size is recommended over Cohen’s *d* when sample sizes are < 50, as was the case in our study. An effect size of 0.5 represents a medium magnitude effect, and > 0.8 a large magnitude effect. Effect sizes > 1.0 are considered to represent about a 55% non-overlap, or dissimilarity of taxa, > 2.0 represents about 80% non-overlap or dissimilarity of taxonomic communities, > 3 represents more than 90% non-overlap or dissimilarity [57]. In addition, Mann-Whitney tests were conducted, comparing the significance of the differences in MPS of genera with a MPS values > 1.0% in one or more of the habitats studied, either in Pre or Post-Hurricane soil samples. We used a cutoff of *p* values < 0.05 to suggest significant differences between MPS values. These genera were then placed into one of the previously identified functional groups associated with the C and N cycle, as appropriate. The magnitude of the differences in the MPS values of these genera were also assessed by calculating the Hedge’s *g* Effect Size. To address question 2, the mean values of the soil temperature, % Water, TOC, Biomass C, Biomass C/TOC, TN, NO_3_^-^, and NH_4_^+^ were examined by one-way analysis of variance (ANOVA) followed by Tukey’s HSD or Dunnett’s T3 post-*hoc* tests, as appropriate, in SPSS (v.25, Armonk, NY, USA) to determine if there were differences in the means between the Pre- and Post-Hurricane soils for each habitat type. Prior to ANOVA, the Levene’s test was performed in SPSS to determine homogeneity of the variances of the data, and the Shapiro-Wilk’s test was performed in SPSS to determine normality of all the data. All data had *p* values > 0.05, suggesting the use of ANOVA was appropriate. To address question 3, a distance-based linear model (DistLM) permutation test was implemented using PERMANOVA+ to determine if there were soil C and/or N-cycle variables that were significant predictors of the multivariate patterns of the soil bacterial and/or fungal community compositions associated with the different soil samples. We used the fourth root transformed resemblance Bray-Curtis matrices of the taxonomic data as response variables, the log (x + 1) transformed C and N data converted into Euclidean matrices as the predictor variables, and a step-wise selection process along with an AICc (Akaike's Information Criterion Corrected) selection criterion and 9,999 permutations [55]. The AICc criterion is applied to handle situations where the number of samples (N) is small relative to the number (v) of predictor variables, where N/v < 40, as per Anderson et al. [55], and as was the case in this study.

## Results

### Differences in abiotic properties

The % Water, Biomass C, Biomass C/TOC, NH_4_^+^ and NO_3_^-^ were significantly greater, and the TN significantly less in the Post-compared to the Pre-Hurricane soils of all three forest habitats, and the TOC levels were also significantly less in the Upland and Riparian Forest Post- compared to Pre-Hurricane samples (S1 Table).

### Differences in total bacterial communities before and after the hurricane

The NGS of the soil eDNA identified 1,342,185 bacterial DNA sequences, of which 538,121 could be categorized into 579 different bacterial genera. There were significant differences in the composition of the Total Bacterial communities found before and after the hurricane (Table 1A and 1B) across the three forest types, and the. The magnitude of these was extremely large between the Pre- and Post-hurricane soil samples from all habitats, which was consistent with the CAP analyses (Table 1C) that showed extremely strong differences in the of this community between the Pre- and Post-Hurricane soil samples from all habitats. The richness of this community (Table 2) was also less in the soils of all three forest types after the hurricane, with very large Effect Sizes indicating > 90% dissimilarity in richness before and after the hurricane. There were also significant differences (*p* < 0.05) found the MPS values of 11 different genera in the soils from all habitats before and after the hurricane (Table 3). The large difference in MPS values and the large Hedge’s *g* values for the comparisons suggest these differences are associated with variations in important ecological process outcomes. The CCD/Lignin Degrading and NH_4_^+^ Producing bacterial genera Acidobacteria Groups 1, 2, 3 and Verrucomicrobia Spartobacteria had far greater MPS values in the Post- than the Pre-Hurricane soils of all forest habitats, with large Effect Sizes indicating > 90% dissimilarity in these MPS values. Conversely, the MPS levels of the CCD/Lignin Degrading genera *Isosphaera*, *Koribacter*, and *Solibacter*, and the NH_4_^+^ Producing genera *Solibacter* and *Rhodoplanes* were greater in the Pre- rather than the Post-Hurricane soils in all habitats, with large Effect Sizes indicating > 90% dissimilarity in these MPS values. The MPS levels of the AMO bacteria *Nitrospira* were only moderately greater in the Pre- than the Post-Hurricane soils from all forest habitats, with Effect Sizes indicating only a 55% or 80% dissimilarity in these MPS values. The DistLM analysis (Table 4) showed the level of TOC was the best predictor of the Total Bacterial community composition in all soils before the hurricane, explaining 21.42% of the variation in the composition of that community (Pseudo-F = 3.54, *p* = 0.010), and after the hurricane, the best predictors were Biomass C, NH_4_^+^, and NO_3_, explaining 36.67% of that community’s variation in composition (Pseudo-F = 2.56, *p* = 0.045).

**Table 1:** 1a. The mean proportion of DNA sequences (MPS) of the CCD/lignin degrading, the NH4+producing, and the AMO bacterial genera, and the CCD/WRT/lignin degrading and ECM fungal genera in soils before and after Hurricane Otto in Lowland, Upland and Riparian Forest plots within the Maquenque National Wildlife Refuge in Costa Rica. 1b. The results of PERMANOVA tests on the differences in the MPS values of these functional groups and the Total Bacterial and Total Fungal genera in the forests before (2016) and after (2017) the hurricane (Pseudo-F values and *p* values given). 1c) Results of the Canonical Analysis of Principal Coordinates (CAP) assessment of the differences in the patterns of the community compositions (R^2^ and *p* values are given).

1a) Bacterial and Fungal Functional Group MPS values							
	MPS of Bacterial Functional Groups			MPS of Fungal Functional Groups			
Soil Samples Types	CCD/Lignin	NH_4_^+^ Producers	AMO	CCD/WRT/Lignin	ECM		
Lowland Pre-Hurricane Samples	34.0 ± 5.1	20.7 ±4.3	8.7 ± 2.4	33.4 ± 6.9	0.9 ±0.7		
Lowland Post-Hurricane Samples	83.6 ± 12.1	86.5 ± 12.3	10.6 ± 2.7	48.3 ± 7.2	1.9 ± 0.10		
Upland Pre-Hurricane Samples	36.61 ± 3.4	23.5 ± 4.4	9.5 ±2.3	16.8 ± 7.7	2.6 ± 0.6		
Upland Post-Hurricane Samples	79.1 ± 10.8	85.1 ± 12.1	11.3 ± 2.1	24.4 ± 5.0	20.3 ± 5.4		
Riparian Post-Hurricane Samples	33.1 ± 7.0	18.3 ± 6.8	9.6 ± 3.0	47.1 ± 3.8	0.8 ± 0.2		
Riparian Pre-Hurricane Samples	82.3 ± 11.1	84.8 ± 11.4	11.7 ± 3.1	55.5 ± 4.6	6.5 ± 1.4		
1b) PERMANOVA of Total and Functional Group Microbe MPS Values							
	Total Bacterial Genera	Total Fungal Genera	CCD/Lig Bacteria	NH4+ Bacteria	AMO Bacteria	CCD/WRT/Lig Fungi	ECM Fungi
Habitat Comparisons	Pseudo-F (*p* value)	Pseudo-F (*p* value)	Pseudo-F (*p* value)	Pseudo-F (*p* value)	Pseudo-F (*p* value)	Pseudo-F (*p* value)	Pseudo-F (*p* value)
Pre- to Post-Hurricane							
Lowland 2016 to 2017	**327.2 (0.007)**	**2.16 (0.061)**	**369.4 (0.007)**	**299.0 (0.008)**	**4.21 (0.015)**	**2.31 (0.052)**	**2.18 (0.009)**
Upland 2016 to 2017	**202.7 (0.009)**	**4.22 (0.009)**	**174.5 (0.009)**	**205.3 (0.008)**	**2.51 (0.036)**	**6.40 (0.007)**	**2.38 (0.015)**
Riparian 2016 to 2017	**185.7 (0.007)**	**2.30 (0.048)**	**262.3 (0.007)**	**127.4 (0.010)**	Not Sig (*p* = 0.119)	**2.47 (0.0004)**	**2.71 (0.007)**
1c) Results of CAP Analyses							
	Total Bacterial Genera	Total Fungal Genera	CCD/Lig Bacteria	NH4+ Bacteria	AMO Bacteria	CCD/WRT/Lig Fungi	ECM Fungi
Habitat Comparisons	CAP R^2^ (*p* value)	CAP R^2^ (*p* value)	CAP R^2^ (*p* value)	CAP R^2^ (*p* value)	CAP R^2^ (*p* value)	CAP R^2^ (*p* value)	CAP R^2^ (*p* value)
Pre- to Post-Hurricane							
Lowland 2016 to 2017	**0.998 (0.0001)**	**0.630 (0.0001)**	**0.989 (0.0001)**	**0.998 (0.0004)**	**0.848 (0.0001)**	**0.417 (0.0004)**	**0.975 (0.018)**
Upland 2016 to 2017	**0.998 (0.0001)**	**0.906 (0.0001)**	**0.989 (0.0001)**	**0.998 (0.0001)**	**0.302 (0.0001)**	**0.884 (0.0004)**	**0.975 (0.018)**
Riparian 2016 to 2017	**0.998 (0.0001)**	**0.630 (0.006)**	**0.989 (0.0001)**	**0.998 (0.0001)**	**0.848 (0.0001)**	**0.639 (0.0004)**	**0.975 (0.018)**

**Table 2:** The mean richness (± standard deviation) of the Total Bacterial and Total Fungal genera, and the genera from the CCD/lignin degrading, NH4+ producing, and AMO bacterial groups, and the genera from the CCD/WRT/lignin degrading, and ECM fungal groups in the soils of the Lowland, Upland and Riparian Forests within the Maquenque National Wildlife Refuge in the Northern Zone of Costa Rica before (Pre-) and after (Post-) Hurricane Otto hit the forests. The differences in means is shown by the PERMANOVA Pseudo-F value, with *p* values, and the Hedge’s *g* Effect Size values for the comparisons in parentheses. Significant differences are high-lighted in bold.

Microbial Group Richness in Soils Pre- and Post-Hurricane							
Soil Samples Types	Total Bacteria	CCD/Lignin	NH_4_^+^ Producer	AMO Bacteria	Total Fungi	WRT/CCD/Lig Fungi	ECM Fungi
Lowland Pre-Hurricane Soils	30.27 ± 4.29	11.84 ± 1.19	7.24 ± 0.82	3.17 ± 0.32	20.67 ± 4.22	8.39 ± 1.79	4.55 ± 0.71
Lowland Post-Hurricane Soils	14.59± 1.94	6.69 ± 0.44	4.04 ± 0.32	2.39 ± 0.63	4.65 ± 2.13	2.10 ± 1.24	0.14 ± 0.08
Upland Pre-Hurricane Soils	32.01 ± 4.40	11.85 ± 0.78	7.29 ± 0.84	3.12 ± 0.36	19.67 ± 2.72	9.19 ± 1.67	4.19 ± 0.72
Upland Post-Hurricane Soils	16.63 ± 3.81	7.43 ± 1.53	4.42 ± 0.60	2.26 ± 0.33	5.69 ± 4.56	3.75 ± 0.91	0.43 ± 0.04
Riparian Pre-Hurricane Soils	29.96 ± 3.49	11.92 ± 0.74	7.95 ± 0.83	2.84 ± 0.17	18.85 ± 4.56	6.51 ± 1.54	4.46 ± 0.50
Riparian Post-Hurricane Soils	15.16 ± 2.91	6.78 ± 1.07	4.24 ± 0.61	2.80 ± 0.81	4.82 ± 0.58	2.11 ± 0.20	0.92 ± 0.17
PERMANOVA of Microbial Group Richness							
	Total Bacteria	CCD/Lig Bacteria	NH4+ Bacteria	AMO Bacteria	Total Fungi	WRT/CCD/Lig Fungi	ECM Fungi
Habitat Comparisons	Pseudo-F (p; *g* value)	Pseudo-F (p; *g* value)	Pseudo-F (p; *g* value)	Pseudo-F (p; *g* value)	Pseudo-F (p; *g* value)	Pseudo-F (p; *g* value)	Pseudo-F (p; *g* value)
Lowland 2016 to 2017	**53.14 (0.009; 4.71)**	**64.16 (0.001; 5.74)**	**118.59 (0.008; 5.14)**	**2.76 (0.053; 1.56)**	**22.36 (0.008; 4.79)**	**17.53 (0.008; 4.09)**	**101.10 (0.008; 8.73)**
Upland 2016 to 2017	**26.94 (0.008; 3.74)**	**41.34 (0.008; 3.64)**	**55.95 (0.007; 3.93)**	**4.69 (0.009; 2.49)**	**70.59 (0.009; 3.72)**	**29.38 (0.008; 4.05)**	**225.36 (0.009; 7.37)**
Riparian 2016 to 2017	**35.16 (0.009; 4.61)**	**33.99 (0.007; 5.59)**	**89.87 (0.006; 5.59)**	Not Sig (p = 0.176)	**37.62 (0.008; 4.23)**	**56.57 (0.009; 4.01)**	**217.87 (0.007; 9.45)**

**Table 3:** Results of the Mann–Whitney analyses showing the significant differences (p < 0.05) in the mean proportion of sequences per sample (MPS) of the genera that were present at > 1% MPS in the Lowland, Upland and Riparian Forest soils, before (Pre-Otto) and after (Post-Otto) Hurricane Otto hit the forests in November 2016. The mean MPS ± the standard deviation, the Hedge’s *g* Effect Size for the different comparisons, and the proposed functions are given. Significantly greater MPS values are high-lighted in bold.

Significant Differences in		Lowland Forest Average MPS			Upland Forest Average MPS			Riparian Forest Average MPS		
Bacterial Genera (p < 0.05)	Functions	Pre-Otto	Post-Otto	Hedge's *d*	Pre-Otto	Post-Otto	Hedge's *d*	Pre-Otto	Post-Otto	Hedge's *d*
Acidobacteria Groups 1, 2, 3	CCD/Lignin, NH4+ Producers	0.40 ± 0.12	**71.12 ± 9.20**	10.87	0.45±0.09	**65.42 ± 2.64**	34.78	0.31 ± 0.19	**72.71 ± 1.74**	58.51
Verrucomicrobia Spartobacteria	CCD/Lignin, NH4+ Producers	0.01 ± 0.01	**3.53 ± 0.82**	6.07	0.02 ± 0.01	**5.35 ± 1.79**	4.21	0.02 ± 0.02	**3.16 ± 1.07**	4.15
Isosphaera	CCD/Lignin	**5.95 ± 0.96**	0.24 ± 0.03	8.41	**1.41 ± 0.31**	0.25 ± 0.22	4.32	**3.01 ± 0.72**	0.35 ± 0.24	4.96
Koribacter	CCD/Lignin	**20.52 ± 2.95**	0.79 ± 0.16	9.44	**23.39 ± 2.27**	0.71 ± 0.29	14.02	**24.33 ± 4.86**	1.30 ± 0.54	6.66
Ktedonobacter	unknown	**8.12 ± 1.02**	0.22 ± 0.08	10.92	**5.89 ± 1.07**	0.26 ± 0.07	7.43	**5.73 ± 1.69**	0.08 ± 0.06	4.73
Nitrospira	AMO	**6.07 ± 1.97**	3.28 ± 0.91	1.82	**5.23 ± 1.10**	3.54 ± 1.62	1.22	**8.54 ± 2.64**	5.06 ± 2.44	1.37
Rhodoplanes	NH4+ Producers	**14.23 ± 2.12**	0.12 ± 0.02	9.41	**16.75 ± 2.30**	0.10 ± 0.07	10.23	**12.69 ± 3.81**	0.01 ± 0.01	4.71
Solibacter	CCD/Lignin, NH4+ Producers	**25.39 ± 2.25**	2.34 ± 0.74	8.42	**28.77 ± 1.30**	2.06 ± 0.12	28.94	**25.78 ± 3.05**	2.36 ± 0.39	8.65
Thermogemmatispora	unknown	**4.33 ± 0.53**	0.34 ± 0.08	10.53	**4.44 ± 0.77**	0.06 ± 0.05	8.03	**5.10 ± 2.14**	0.03 ± 0.04	3.35
Significant Differences in		Lowland Forest Average MPS			Upland Forest Average MPS			Riparian Forest Average MPS		
Fungal Genera (p < 0.05)	Functions	Pre-Otto	Post-Otto	Hedge's *g*	Pre-Otto	Post-Otto	Hedge's *g*	Pre-Otto	Post-Otto	Hedge's *g*
Calonectria	CCD/WRT/Lignin	**1.23 ± 0.59**	0.37 ± 0.33	1.79	**0.88 ± 0.43**	0.51 ± 0.48	0.81	**0.95 ± 0.29**	0.53 ± 0.43	1.14
Clavaria	ECM	0.05 ± 0.02	0.04 ± 0.03	ND	0.07 ± 0.02	**11.42 ± 7.57**	2.12	0.06 ± 0.03	0.05 ± 0.03	ND
Cryptococcus	CCD/WRT/Lignin	12.88 ± 3.12	**17.51 ± 8.56**	0.81	1.26 ± 0.52	**6.87 ± 3.75**	2.1	10.89 ± 3.41	**15.88 ± 5.69**	1.06
Geotrichum	CCD/WRT/Lignin	2.73 ± 0.43	**25.71 ± 6.74**	4.82	1.02 ± 0.76	**4.63 ± 1.50**	3.04	14.29 ± 5.26	**23.37 ± 10.47**	1.11
Gliocladiopsis	CCD/WRT/Lignin	1.86 ± 1.09	**4.64 ± 2.56**	1.41	0.73 ± 0.38	**3.31 ± 1.23**	2.83	2.26 ± 1.36	**10.62 ± 5.54**	2.07
Hygrocybe	ECM	0.040 ± 0.06	**0.84 ± 1.25**	0.9	0.13 ± 0.03	**8.87 ± 3.98**	3.1	0.15 ± 0.03	**6.43 ± 2.44**	3.64
Mortierella	CCD/WRT/Lignin	4.78 ± 0.58	**12.37 ± 6.87**	1.56	8.88 ± 5.36	**34.17 ± 16.32**	2.08	1.24 ± 1.08	**11.39 ± 8.31**	1.71
Myxocephala	unknown	**3.18 ± 1.97**	1.22 ± 1.05	1.24	**3.57 ± 1.55**	1.17 ± 0.77	1.96	**1.72 ± 0.56**	1.05 ± 0.68	1.08
Trichoderma	CCD/WRT/Lignin	**3.15 ± 1.81**	0.78 ± 0.59	1.76	**3.12 ± 0.96**	0.53 ± 0.45	3.45	**2.27 ± 1.80**	0.27 ± 0.35	1.54
Trichosporon	unknown	**41.3 ± 15.70**	24.13 ± 11.86	1.23	**46.48 ± 16.39**	18.34 ± 7.00	2.23	**35.08 ± 18.99**	16.06 ± 6.67	1.33
Xylaria	CCD/WRT/Lignin	**1.65 ± 1.11**	0.72 ± 0.65	1.02	**2.40 ± 1.42**	0.28 ± 0.25	2.08	**2.69 ± 1.17**	0.57 ± 0.28	2.49

**Table 4:** The distance-based linear modeling (DistLM) sequential tests identifying the environmental variables that best predict the variation in the patterns in the composition of the Total Bacterial and the Total Fungal Genera communities, in soils from 3 different forest types in the Maquenque National Wildlife Refuge in the Northern Zone of Costa Rica, before and after Hurricane Otto. The variables considered were soil temperature, % Water, total organic carbon (TOC), total nitrogen (TN), nitrate (NO_3_^-^), ammonium (NH_4_^+^), Biomass C, and Biomass C/TOC. Stepwise sequential tests were used, following AICc criteria. The Pseudo-F and *p* values are given along with the cumulative proportion of the total variation (Cv Prop) for the best predictive model.

Total Bacterial Genera Before Hurricane Otto 2016				
Sequential Tests	AICc	Pseudo-F	*p*	Cv Prop
TOC	69.15	3.54	0.010	21.4%
Total Bacterial Genera After Hurricane Otto 2017				
Sequential Tests	AICc	Pseudo-F	*p*	Cv Prop
Biomass C, NH_4_^+^, NO_3_^-^	71.60	2.56	0.045	36.7%
Total Fungal Pre-Hurricane Before Hurricane Otto				
Sequential Tests	AICc	Pseudo-F	*p*	Cv Prop
TOC, % Water	103.49	3.96	0.009	37.5%
Total Fungal Post-Hurricane After Hurricane Otto				
Sequential Tests	AICc	Pseudo-F	*p*	Cv Prop
Biomass C, NO_3_^-^, % Water	123.53	3.04	0.034	41.6%

### Differences in total fungal communities before and after the hurricane

The NGS of the soil eDNA identified 1,114,085 fungal DNA sequences, of which 433,845 were identified into 308 different fungal genera. Significant differences were observed in the composition of the Total Fungal communities in the soils across all three forest types before and after the hurricane (Table 1A and 1B). The magnitude of these differences was far less than that of the Total Bacterial communities, however, the CAP analyses (Table 1C) did show strong differences in the community compositions between the Upland Forest soil samples, and moderate differences between the Lowland Forest and the Riparian Forest soils Pre- and Post-Hurricane. Similar to the bacterial results, the richness of these genera was less (Table 2) in the soils before compared to after the hurricane in all three forest habitats, with large Effect Sizes indicating > 90% dissimilarity in richness. There were significant differences in MPS values of 11 genera (*p* < 0.05) between the Pre- and Post-Hurricane soils (Table 3). The MPS values of the CCD/WRT/Lignin genera *Calonectria*, *Trichoderma*, and *Xylaria* were greater in all forest habitat Pre- compared to Post-Hurricane soils, while the genera within the same functional group *Cryptococcus*, *Geotrichum*, *Gliocladiopsis* and *Mortierella* were greater in the Post- compared to the Pre-Hurricane soils in all forest habitats, with large Effect Sizes indicating between about 55% and >90% dissimilarity in the MPS values for all these genera before and after the hurricane. The MPS levels of the ECM genus *Hygrocybe* were greater in the Post-compared to the Pre-Hurricane soils of all three forest types, while the MPS level of the ECM genus *Clavaria* was only greater in the Upland Forest Post- compared to Pre-Hurricane soils, with large Effect Sizes indicating about 55% to > 90% dissimilarity in the MPS values for both these genera. The MPS of the genus *Trichosporon* was large in all forest habitat soils, but significantly greater in the Pre- compared to the Post-Hurricane soils across all three forest habitats, with large Effect Sizes that indicated > 90% dissimilarity in these MPS values The DistLM analysis (Table 4) showed the levels of TOC and % Water were the best predictor of the Total Fungal community composition in all soils before the hurricane, explaining 37.50% of the variation in the composition of that community (Pseudo-F = 3.96, *p* = 0.009), and after the hurricane, the best predictors were Biomass C, NO_3_^-^, and % Water, explaining 41.6% of that community’s variation in composition (Pseudo-F = 3.04, *p* = 0.034).

### Differences in the functional group communities of bacteria before and after the hurricane

From the 579 bacterial genera identified by NGS analyses of the eDNA, there were 132 genera placed in the CCD/Lignin Degrading group, 79 in the NH_4_^+^ Producing group, and 28 in the AMO group. There were significant differences found in the MPS values of these functional group communities in the soils before and after the hurricane (Table 1A and 1B). The MPS values for the CCD/Lignin Degrading and NH_4_^+^ Producing community compositions were far greater in the Pre- compared to the Post-Hurricane soils from all three forest habitats, while those of the AMO bacterial community were greater in the Upland and Lowland Forest soils after compared to before the Hurricane, but the magnitude of these differences was far less than for the other two functional groups. The CAP analyses (Table 1C) showed strong differences in the compositions of the CCD/Lignin Degrading and the NH_4_^+^ Producing bacterial communities between the Pre- and Post-Hurricane soils from all three forest habitats, and somewhat decreased levels of difference in the AMO bacterial community composition between the Pre- and Post-Hurricane soils from all three habitats. The richness (Table 2) of the CCD/Lignin Degrading Bacteria and NH_4_^+^ Producing bacterial communities was greater in the Post- than the Pre-Hurricane soils for all forests, with large Effect Sizes indicating > 90% dissimilarity in community richness. The richness of the AMO bacterial communities was also significantly greater, although at a greatly reduced magnitude, in the Pre-compared to Post-Hurricane soils for the Lowland and Upland Forests, with large Effect Sizes indicating between 55% and 80% dissimilarity in community richness.

### Differences in functional group communities of fungi before and after the hurricane

From the 308 fungal genera identified by NGS analyses of the eDNA, 64 genera were placed in the CCD/WRT/Lignin Degrading group and 32 in the ECM group. The composition of both of these functional group communities were different between the Pre- and Post-Hurricane soils of all three forest types. In addition, the MPS values of the CCD/WRT/Lignin Degrading and the ECM fungal genera were greater in the Post-Hurricane than Pre-Hurricane soils for the different forest types. The CAP analyses (Table 2) showed significant differences in the composition of the CCD/WRT/Lignin degrading fungal communities between the Pre- and Post-Hurricane soils for all three forest habitats. Specifically, strong differences in the group were found between the Pre- and Post-Hurricane samples in the Uplands Forest, moderate differences for this group were found before and after the hurricane in the Riparian Forest soils, and weak differences were found in Lowland Forest soils between the Pre- and Post-Hurricane samples. In addition, the CAP analyses showed very strong differences in the community composition of the ECM genera between the Pre- and Post-Hurricane soils for all three forest types. The patterns of the fungal richness (Table 4) were similar to that found for the bacterial communities, in that, although the MPS values were greater, the richness of CCD/WRT/Lignin Degrading and ECM fungal communities decreased in the Post-Hurricane compared to Pre-Hurricane soils from all three forest habitats. The Effect Sizes were also very large, indicating > 90% dissimilarity in the richness of these communities before and after the hurricane.

## Discussion

Increased amounts of leaf and woody material deposited onto forest floors from disturbances, and the subsequent increased precipitation reaching the forest floor, are thought to select for soil microbial communities to degrade the deposited material, However, these microbes would require an increase in usable C and N for the production of degradative enzymes to decompose and process these forest floor materials [22]. Given that tropical forest hurricanes rapidly deposit large amounts of leaf litter and woody debris from the canopy to the forest floor [4–7], followed by an increased amount of precipitation reaching the soils due to the loss of the protective canopy cover [9, 24, 28], it is likely this would select for microbial communities involved in the decay, decomposition, and processing of the deposited canopy materials [8, 10, 12, 13]. These microbial metabolic processes would require more usable organic C, NO_3_^-^, and both inorganic and organic forms of NH_4_^+^ for production of an extensive array of enzymes needed for this decomposition [4, 6, 8–10, 14, 22–29]. Although it is thought that such changes in the tropical soil microbial community are critical to decay canopy material after disturbances, there is still very little known about how hurricanes specifically influence these soil biotic communities [14, 24, 25].

This current project partially addresses this knowledge gap, as it represents a serendipitous opportunity to measure the effects that a naturally occurring hurricane had on the soil biotic and abiotic components in a tropical forest within the same forest floor plots before and 7 months after Hurricane Otto damaged the forests. In this work, we provide evidence to confirm our predictions that there would be post-hurricane increases in the soil water, inorganic N, Biomass C, and Biomass C/TOC, and increases in the MPS values, and changes in the community composition of the general soil bacterial and fungal communities, the specific bacterial groups associated with the N cycle, and the bacterial and fungal groups associated with decomposition of more complex forms of organic C, 7 months after Hurricane Otto hit these jungle sites. The patterns of changes observed in the soil water, C, N, and Biomass C metrics Post- compared to Pre-Hurricane soils are consistent with the literature, and the patterns of changes observed in the soils after the hurricane within the microbial communities suggest the critical role that specialized microbial functional groups play in the early phases of processing of the deposited canopy material during soil ecosystem recovery following a hurricane.

There have been studies that have shown the deposition of canopy material onto the tropical forest floor during a hurricane simulation results in nutrient pulses of the more labile forms of C and N derived from the canopy-deposited younger leaf litter for about the first several years [4, 10, 14, 26–28]. This material is quickly processed within the first year or so [7, 11, 19], and is followed by a period of decay of the deposited woody debris, resulting in a slower rate of processing activities [9, 11, 17–20]. All these metabolic activities would be associated with the types of increases in inorganic N and Biomass C, and changes in the microbial communities that we observed in the forest soils 7 months after Hurricane Otto hit these forests [13, 40, 58–62]. If the recovery process in these forest soils follows the trends from the literature, then we would expect to start to see decreases in C-use efficiency (Biomass C/TOC) and shifts towards more wood rot and lignin decaying fungi, along with decreases in the inorganic N levels, and decreases in the N-cycle-associated bacterial genera over time. To monitor for these patterns, we are conducting a long-term study in the plots, collecting data annually to analyze for subsequent patterns that emerge.

In the current study, in all Post- compared to Pre-Hurricane soils, there were increased MPS levels, significant differences in the community compositions, but significant reductions in the richness of all five specialized functional group communities of bacteria and fungi, as well as significant reductions in richness of the Total Bacterial and Total Fungal communities. This suggests the microbial communities changed from more diverse to less diverse, and perhaps became more specialized and dominant communities in the Post- compared to the Pre- Hurricane soils, as they were likely selected for enhanced decay, decomposition, and processing of the excessive canopy material deposited on the forest floor during the hurricane [22]. However, although such changes in the microbial community would be expected, they have not been well-characterized for tropical forests soils after a hurricane [14, 24, 29].

Similar to our work, previous studies have shown that forest disturbances were associated with increases in the abundance of, and shifts in diversity away from the more generalist microbes and more towards the more specialized opportunist or “r-strategist” soil microbes, such as the complex C degrading and N-cycle microbes involved in decomposition of the forest debris [22, 63, 64]. It is important to note that these patterns of microbial community change post-disturbance in forests are consistent with Odum’s theory that young and disturbed ecosystems will have a lower ratio of K-strategists to r-strategists than mature and undisturbed ecosystems [65]. The patterns we observed were consistent with these other tropical forest post-disturbance results, potentially providing predictive insights into trends to watch for during the recovery of these forests.

It was of interest to note that there were far greater increases in bacterial compared to fungal community MPS values in the Post- compared to Pre-Hurricane soils. Elevated levels of soil moisture have been associated with increases in the activities of N-fixing, AMO, N-mineralizing bacterial groups [66–70], similar to our observations of, greater levels of MPS of AMO and NH_4_^+^ Producing bacteria being associated with greater levels of %Water and inorganic N in the Post- compared to Pre- Hurricane soils. Greater levels of soil moisture in post-disturbance forest soils have also been associated with increased rates of soil decomposition and soil biomass C generation, presumably selecting for bacteria involved in these processes [66, 71–73]. We observed increased % Water in the soil. and hurricane-induced canopy material on the forest floor in all Post-Hurricane forests appearing to select for large increases in level of MPS of the CCD/Lignin bacteria that were associated with the greater levels of Biomass C, and Biomass C/TOC observed in all soils. In contrast, only moderate increases in the different fungal community MPS levels were observed within all forest types Post- compared to the Pre-Hurricane soils in our study, which likely shows that these different fungal groups, important in decomposition of complex organic C, tend to develop later in recovery due to their requirement for the N and C components produced by decomposing bacteria earlier in the recovery process [19–22]. However, this variation in magnitude of difference in MPS levels between bacteria and fungi in soils after the hurricane could also be a function of fungal compared to bacterial communities being more sensitive to such excessive increases in soil moisture as observed in the soils after the hurricane [66, 74, 75]. Clearly, then, at least within the “Immediate Effects” period Post-Hurricane in these forests, the soil bacterial CCD/Lignin communities were more influenced than the fungal CCD/WRT/Lignin communities, suggesting than bacterial communities he bacterial groups is more rapidly changing due to the disturbance.

The observed increases in MPS values and decreases in richness levels for all five functional group communities were also associated with significant changes in specific genera between the Pre-and Post-Hurricane soils, which may represent critical genera important in the recovery process. Specifically, the MPS levels of *Isosphaera*, *Koribacter*, and *Solibacter* associated with CCD/Lignin Degradation, and *Solibacter* and *Rhodoplanes* associated with NH_4_^+^ Production were far greater (> 90% dissimilarity in MPS values) in the Pre- compared to the Post-Hurricane soils in all three forest habitats. This suggests that these two groups of genera are extremely important in the decomposition of organic material and the generation of NH_4_^+^ needed for enzyme production and cellular biosynthesis in these forest soils under the undisturbed conditions in the Pre-Hurricane soils. However, the MPS of these genera were almost completely replaced in the Post-Hurricane soils of all three forest habitats by the bacterial genera classified as Acidobacteria Groups 1, 2, 3 and Verrucomicrobia Spartobacteria, both of which have CCD/Lignin Degrading and NH_4_^+^ Producing capabilities, resulting in > 90% dissimilarity in the MPS levels of these genera in the soils Post- compared to Pre-Hurricane. This suggests that these genera are being selected for in the Post-Hurricane soils as they may be of critical importance in the decay and decomposition of the canopy material, and the generation of NH_4_^+^ needed in order for this decay to happen, and may be important indicators of early recovery in these soils. Studies have shown a positive correlation between the abundance of Acidobacteria genera and levels of available C resources in soils [76–79], and that Acidobacteria appear to be very important in recovering soils as they enhance C and N cycling processes, and are beneficial to plant growth after extreme ecological disturbances [78, 80, 81]. Similarly, genera within the Verrucomicrobia have been shown to be correlated with C and N levels that increase during litter decay in more moist tropical soils, degrade more recalcitrant organic C materials, and are important in metabolizing plant C compounds, and are important in recovery of forest soils from disturbance [79, 81–83]. The increased MPS and reduced richness of both of these groups of bacterial genera suggests they are becoming more dominant and are playing similar roles in the Post-Hurricane soils examined in this study. The changes in the MPS values between the CCD/Lignin Degrading and NH_4_^+^ Producing genera suggests that taxonomic switching of functionally redundant bacterial genera may be occurring in these soils after the hurricane, and, consistent with the literature [75–84], selection of the Acidobacteria Groups 1, 2, and 3 and Verrucomicrobia Spartobacteria genera may indicate that these genera may be more effective at decaying and processing the deposited canopy material post-hurricane. Changes in these groups of genera should be monitored over time to determine if there is a shift back to the original bacterial composition patterns pre-Hurricane over time, which would support the idea that the genera appearing in the Post-Hurricane soils are more efficient opportunists that replaced the more steady-state genera present in the Pre-Hurricane soils. If that is the case, then it is possible that the Acidobacteria and Verrucomicrobia genera may serve as indicators of soil ecosystem recovery following extreme forest ecosystem disturbance.

Changes in the composition of the soil fungal communities associated with wood rot, lignin degradation, degradation of other complex C compounds, and associated changes in Biomass C and C-use efficiency, have been observed in soils following various types of forest disturbances [20, 38, 84], and as such, these should be considered as possible indicators of soil ecosystem recovery. In our study, we found greater MPS values of the CCD/WRT/Lignin genera *Calonectria*, *Trichoderma*, and *Xylaria* in the Pre-compared to the Post-Hurricane soils, and greater MPS values the CCD/WRT/Lignin genera *Cryptococcus*, *Geotrichum*, *Gliocladiopsis* and *Mortierella* in the Post- compared to the Pre-Hurricane soils in all three forest habitats. These shifts in fungal composition may suggest that taxonomic switching is also occurring in the composition of functionally redundant fungal CCD/WRT/Lignin degrading genera, selecting for genera that are most efficiently decaying the deposited canopy materials. Similar to the bacterial genera mentioned above, the composition of these fungal genera should be monitored over time to determine if there is a shift back to the original fungal composition patterns Pre-Hurricane, as this, too, would suggest these genera appearing in the Post-Hurricane soils are more efficient at decay and decomposition, and could represent indicators of soil ecosystem recovery following extreme forest ecosystem disturbance.

The MPS of the fungal genus *Trichosporon* was large in all forest habitat soils, but was much greater in the Pre- compared to the Post-Hurricane soils across all three forest habitats. The function of this genus is unclear, although several have suggested it may have the capacity to decompose polyaromatic organic C compounds and lignin, indicating it could be considered part of the CCD/WRT/Lignin degrading fungal functional group [85, 86]. Given this, and the large MPS values of this genus both before and after the hurricane in all forest type soils, it may be that *Trichosporon* is important in decay of complex organic C and lignin both in the undisturbed and the disturbed forest soils after the hurricane.

The ECM MPS values also increased with decreased levels of richness in the Post- compared to the Pre-Hurricane soils from all three forest habitats in our study. The MPS of *Hygrocybe* was greater in the Post- compared to the Pre-Hurricane soils samples, and that of *Clavaria* exhibited the opposite pattern. Monitoring of the soils over time may provide more evidence that the former genus may be an indicator of soil recovery, while the latter may be more of an indicator of undisturbed soil conditions in these forests. The ECM fungal genera are known to degrade complex organic C compounds, more recalcitrant C such as lignin, are associated with plant materials in soils and on forest floors for C and N, and provide both organic C and N for use in biomass development [20, 54, 87–90]. The increases in the MPS of this group, along with the increases in Biomass C, Biomass C/TOC, and the NH_4_^+^ levels in the Post- compared to the Pre-Hurricane soils from all forest habitats supports these ideas on the role of ECMs in forest soils, and suggests that changes in the structure of the ECM community may also serve as another indicator of soil recovery after the hurricane, as this group of fungi have also been shown to be important in enhancing plant communities and recovery after disturbances [4, 54, 91, 92].

The changes observed in the bacterial and fungal community structures within the soils before and after the hurricane in this study were significantly linked with different soil C and N components that are consistent with the critical roles these microbes are known to play in the C and N cycle dynamics [59, 60, 62, 65–72, 74]. Specifically, the DistLM analyses showed that the TOC levels and the TOC levels with % Water were the best predictors of the variations in composition of the Total Bacterial and Total Fungal communities, respectively, in the Pre-Hurricane soils, while the best predictors of the variations in the composition of the Total Bacterial and Total Fungal community structures in the Post-Hurricane soils were significantly linked with the inorganic N and Biomass C components (i.e., Biomass C, NH_4_^+^, and NO_3_^-^) that are associated with the role of all five functional groups of microbes in soil recovery. These results suggest that increases in the levels of microbial processing of the canopy material is occurring in the Post- compared to the Pre-Hurricane samples, and is associated with the increases in the MPS values and decreases in richness of the CCD/Lignin degrading, NH_4_^+^ Producing and AMO bacterial genera, and the CCD/WRT/Lignin degrading and ECM fungal genera in the Post-Hurricane soil samples in all three forest types, as well as with the changes in the MPS of the 11 bacterial and 11fungal genera that occurred between the Pre- and Post-Hurricane soils.

It is not surprising that the hurricane-induced deposition of leaf litter and woody debris would be associated with the types of alterations we observed in the microbial populations, and C, N, and Biomass C metrics in the soils after the hurricane. Others have suggested that, presumably, populations of the lignin degrading and complex C degrading bacteria and fungi, the wood rot fungi, the decomposer role of the ECM fungi [19, 20, 93–96], and many of the N cycle-associated bacteria ([18, 97–101] would all be enhanced following hurricane-deposited canopy material, however, to date, this has not been demonstrated. As these specialized groups would be selected for, then it is also not surprising that the overall microbial richness might temporarily decrease, as these specialized groups would be favored and more dominant during this early recovery phase that Lugo [8] called the “Immediate Effect” period. These microbial community changes in the soils would be expected to be associated with changes in decomposition activities, early shifts towards the breakdown of more labile forms of organic C, increased efficiency of use of soil organic of C and N, and increased production of inorganic N within these soil ecosystems [48, 58–62]. The degree to which these activities occur within the belowground regions of the tropical forests post-hurricane will most certainly influence the rates of recovery of vegetation community diversity and health, and proper forest ecosystem functioning after the disturbance [12, 13], and should be further studied.

## Conclusions

In this current study, we provide the first evidence that specific bacterial and fungal functional groups and genera are changing in soils after a major hurricane hit this region of Costa Rica, and the possible roles these microbes are playing in the C and N cycles. The important project findings suggest that the hurricane-induced deposition of canopy material and subsequent increase in soil water are selecting for certain microbial functional groups and genera critical for organic C decomposition and N cycle processes, which are becoming more both abundant and dominant in the Post-compared to Pre-Hurricane soils. This suggests that these microbial groups are better adapted to processing the hurricane-deposited canopy material, resulting in increases in inorganic N, Biomass C, and Biomass C/TOC levels in the Post- compared to Pre-Hurricane soils, all of which are critical for soil ecosystem recovery and incorporation of C and N component into the local food webs. As well, there also appear to be certain bacterial and fungal genera that may serve as good indicators of well-functioning, undisturbed forest soil ecosystems in the region, and others as good indicators of disturbed and recovering soil ecosystems. These genera should be monitored in local and regional forests following disturbance by extreme weather events, deforestation, other major disturbances, or restoration practices.

Information on the influences that hurricanes, and other extreme weather events, have on both short- and long-term changes in the soil microbial community components linked to the altered C and N processes post-disturbance still remains largely under-studied. Given the likelihood of an increasing frequency of hurricanes (or other extreme tropical weather events), determining how such major tropical forest disturbances influence the composition and functions of the forest soil ecosystems and ecological processes during recovery should be an area of significant interest.

## Supporting information

S1 Appendix (DOCX)

S1 Table (DOCX)
