# Supplementary material for: Increase in abundance and decrease in richness of soil microbes following Hurricane Otto in three primary forest types in the Northern Zone of Costa Rica
Source: PLoS One. 2020 Jul 30;15(7):e0231187. doi: 10.1371/journal.pone.0231187 (PMC7392270; doi:10.1371/journal.pone.0231187)
Supplement: S1 Appendix — (DOCX) [file pone.0231187.s001.docx]

Appendix S.1. Detailed DNA sequencing and bioinformatics processes.

Environmental microbial DNA (eDNA) was extracted from three 0.33g replicate sub-samples for a total of 1g for each soil sample using the MoBio PowerSoil DNA Isolation Kit (MO BIO Laboratories Inc., Carlsbad, CA, USA). Environmental microbial DNA was extracted from three 0.33g replicate sub-samples soil for a total of 1g for each soil sample using the MoBio PowerSoil DNA Isolation Kit (MO BIO Laboratories Inc., Carlsbad, CA, USA). All eDNA was stored at -80^o^C prior to downstream analysis. The concentration and purity (A_260_/A_280_ ratio) of extracted soil eDNA were determined prior to downstream analyses using a NanoDrop 1000 spectrophotometer (ThermoFisher Scientific, Waltham, MA). All methods described below are from McGee et al [1,2]. PCR amplification of eDNA was performed targeting the V3 and V4 of 16S ribosomal RNA gene region for bacteria and archaea [3] and the nuclear internal transcribed spacer (ITS) ribosomal RNA gene region for fungi [4]. One fragment of the 16S gene region was amplified by PCR targeting two non-overlapping variable gene regions v3 (~197 bp) and v4 (~288 bp) using one primer set 16Sv3F 5'-ACTCCTACGGGAGCAGCAG-3' and 16Sv4R 5'-GGACTACARGGTATCTAAT-3' [5]. One fragment of the ITS gene region was amplified by PCR targeting the ITS1 and ITS2 (variable > 500 bp) regions using one primer set, ITS4 5'-TCCTCCGCTTATTGATATGC-3' and ITS1F 5'-CTTGGTCATTTAGAGGAAGTAA-3' [6]. Amplicons were prepared with two steps PCR regime. The first step was performed with the target specific primers. Each PCR amplification contained 2 µL DNA template, 17.5 µL molecular biology grade water, 2.5 µL 10x reaction buffer (200 mM Tris-HCl, 500 mM KCl, pH 8.4), 1 µL 50x MgCl_2_ (50 mM), 0.5 µL dNTPs mix (10 mM), 0.5 µL forward primer (10 mM), 0.5 µL reverse primer (10 mM), and 0.5 µL Invitrogen Platinum Taq polymerase (5 U/µL) in a total volume of 25 µL. The PCR conditions were 95 °C for 5 min; 35 cycles of 94 °C for 40 s, 46 °C for 1 min, and 72 °C for 30 s; and 72 °C for 5 min; and held at 4°C. PCR products were visualized on 1.5% agarose gels to confirm successful amplification by the presence of fluorescent bands under a UV spectrophotometer. PCR products were then puriﬁed using a Qiagen MinElute PCR puriﬁcation kit (Qiagen, Valencia, CA, USA) and eluted in 30 µL of molecular biology grade water. A second PCR step was implemented using the purified 1^st^ PCR product as a template and with Illumina adaptor tailed target specific primers. The 2^nd^ PCR was made following the same protocol as aforementioned except for 30 cycles were used for PCR. All PCRs were done using Eppendorf Mastercycler ep gradient S thermalcyclers and negative control reactions (no DNA template) were included in all experiments. All generated soil amplicons plates were dual indexed and sequenced in several Illumina Miseq runs using a V2 Miseq sequencing kit (500 cycles - 250 × 2)(FC-131-1002 and MS-102-2003).

To characterize the soil bacterial communities, the 16S Illumina generated sequences were paired-end according to the size of the amplicons using SEQPREP software (https://github.com/jstjohn/SeqPrep) requiring a minimum overlap of 25bp and no mismatches and quality filtered and primers trimmed using PRINSEQ v0.20.4 [7] with a minimum of 20 Phred score (99% base call accuracy) with a window of 10, and a step of 5. The passed quality filtered sequences were denoized with USEARCH [8] [9] at 99% similarity and de novo chimeric sequences removed. Subsequent 16S sequence clusters were taxonomically identified using the Ribosomal Database Project (RDP) [10] with a cutoff value of 95%, resulting in operational taxonomic units (OTUs).

To characterize the soil fungal communities, Illumina generated forward and reverse fragments of ITS sequences were analyzed separately. Forward and reverse ITS sequences were quality filtered and primers trimmed using PRINSEQ v0.20.4 [7] with a minimum of 10 Phred score with a window of 10, and a step of 5. The passed quality filtered sequences were denoized with USEARCH [8] [9] at 99% similarity and de novo chimeric sequences removed. Subsequent ITS sequence clusters were identified using the MEGA-BLAST algorithm [11] against a reference library of all ITS sequences downloaded in the GenBank Database with a minimum percent identity of 90, minimum word size of 28, and a minimum *E*-value of 1e-20.

1. McGee KM, Eaton WD, Shokralla S, Hajibabaei M. Determinants of Soil Bacterial and Fungal Community Composition Toward Carbon-Use Efficiency Across Primary and Secondary Forests in a Costa Rican Conservation Area. Microb Ecol. 2018;10: 423. doi:10.1007/s00248-018-1206-0

2. McGee KM, Eaton WD, Porter TM, Shokralla S, Hajibabaei M. Soil microbiomes associated with two dominant Costa Rican tree species, and implications for remediation_ A case study from a Costa Rican conservation area. Appl Soil Ecol. 2019;137: 139–153. doi:10.1016/j.apsoil.2019.02.007

3. Caporaso JG, Lauber CL, Walters WA, Berg-Lyons D, Lozupone CA, Turnbaugh PJ, et al. Global patterns of 16S rRNA diversity at a depth of millions of sequences per sample. Proc Nat Acad Sci. 2011;108: 4516–4522. doi:10.1073/pnas.1000080107

4. Gardes M, Bruns TD. ITS primers with enhanced specificity for basidiomycetes ‐ application to the identification of mycorrhizae and rusts. Mol Ecol. 1993;2: 113–118. doi:10.1111/j.1365-294X.1993.tb00005.x

5. Sundquist A, Bigdeli S, Jalili R, Druzin ML, Waller S, Pullen KM, et al. Bacterial flora-typing with targeted, chip-based Pyrosequencing. BMC Microbiol. 2007;7: 108–11. doi:10.1186/1471-2180-7-108

6. White TJ, Bruns T, a SLP protocols, 1990. Amplification and direct sequencing of fungal ribosomal RNA genes for phylogenetics In: Innis MA, Gelfand DH, Sninsky JJ, White TJ, editors. PCR …. Academic Press San Diego;

7. Schmieder R, Edwards R. Quality control and preprocessing of metagenomic datasets. Bioinformatics. 2011;27: 863–864. doi:10.1093/bioinformatics/btr026

8. Edgar RC. Search and clustering orders of magnitude faster than BLAST. Bioinformatics. 2010;26: 2460–2461. doi:10.1093/bioinformatics/btq461

9. Edgar RC. UNOISE2: improved error-correction for Illumina 16S and ITS amplicon sequencing. bioRxiv. 2016 [cited 27 Aug 2019]. doi:10.1101/081257

10. Wang Q, Garrity GM, Tiedje JM, Cole JR. Naive Bayesian classifier for rapid assignment of rRNA sequences into the new bacterial taxonomy. Appl Environ Microbiol. 2007;73: 5261–5267. doi:10.1128/AEM.00062-07

11. Zhang Z, Schwartz S, Wagner L, Miller W. A greedy algorithm for aligning DNA sequences. J Comput Biol. 2000;7: 203–214. doi:10.1089/10665270050081478
