# Supplementary material for: Increase in abundance and decrease in richness of soil microbes following Hurricane Otto in three primary forest types in the Northern Zone of Costa Rica
Source: PLoS One. 2020 Jul 30;15(7):e0231187. doi: 10.1371/journal.pone.0231187 (PMC7392270; doi:10.1371/journal.pone.0231187)
Supplement: S1 Table — (DOCX) [file pone.0231187.s002.docx]

Table S.1. The mean values of the soil temperature, % water, total organic carbon (TOC), total nitrogen (TN), nitrate (NO_3_^-^), ammonium (NH_4_^+^), Biomass C, and Biomass C/TOC from the 3 different forest types (Lowland, Upland, and Riparian), before and after Hurricane Otto, within the same forested area of the Maquenque National Wildlife Refuge in the Northern Zone of Costa Rica. Significant differences (*p* < 0.05) are indicated by different letters between Pre- and Post-Hurricane paired comparisons (“a” and “b” represents a significant difference).

| Habitat | TOC | Biomass C | Biomass C/TOC | TN | NH4 | NO3 | Temp | % Water |
| --- | --- | --- | --- | --- | --- | --- | --- | --- |
| Lowland Pre-Hurricane | 598.8 ± 23.7^a^ | 410.8 ± 66.6^a^ | 0.5 ± 0.09a | 48.2 ± 1.1^a^ | 1.39 ± 0.21^a^ | 8.6 ± 1.5^a^ | 25.3 ± 0.22^a^ | 62.9 ± 4.46^a^ |
| Lowland Post-Hurricane | 594.2 ± 39.6^a^ | 522.2 ± 69.0^b^ | 0.88 ± 0.11b | 41.5 ± 1.2^b^ | 5.1 ± 1.59^b^ | 11.0 ± 2.0^b^ | 25.6 ± 0.31^a^ | 82.7 ± 6.40^b^ |
| Upland Pre-Hurricane | 668 ± 28.4^a^ | 351.4 ± 48.3^a^ | 0.52 ± 0.05a | 52.8 ± 1.0^a^ | 0.77 ± 0.30^a^ | 18.3 ± 1.6^a^ | 25.9 ± 0.24^a^ | 56.9 ± 3.87^a^ |
| Upland Post-Hurricane | 534.8 ± 27.0^b^ | 629.6 ± 68.6^b^ | 1.09 ± 0.08b | 44.8 ± 1.6^b^ | 2.94 ± 0.28^b^ | 24.6 ± 3.4^b^ | 26.3 ± 0.21^a^ | 75.3 ± 8.70^b^ |
| Riparian Pre-Hurricane | 562.0 ± 29.7^a^ | 427.8 ± 39.3^a^ | 1.05 ± 0.13a | 50.6 ± 1.6^a^ | 0.72 ± 0.18^a^ | 14.0 ± 2.1^a^ | 24.9 ± 0.46^a^ | 64.6 ± 5.80^a^ |
| Riparian Post-Hurricane | 525.2 ± 17.9^b^ | 583.2 ± 60.8^b^ | 1.01 ± 0.09a | 37.0 ± 1.4^b^ | 2.01 ± 0.44^b^ | 17.8 ± 1.8^b^ | 26.3 ± 0.23^a^ | 79.1 ± 11.03^b^ |
|  |  |  |  |  |  |  |  |  |
|  |  |  |  |  |  |  |  |  |
